# Prescription of psychotropic drugs in persons with concurrent mental health and substance use disorders before and during the COVID-19 pandemic in Norway

**DOI:** 10.1192/j.eurpsy.2025.1485

**Published:** 2025-08-26

**Authors:** M. Leonhardt, L. Lien

**Affiliations:** 1 ROPforsk, Innlandet Hospital Trust, Brumunddal; 2 Faculty of Social and Health Sciences, Inland Norway University of Applied Sciences, Elverum, Norway

## Abstract

**Introduction:**

The COVID-19 pandemic has had a profound impact on mental health globally, exacerbating existing mental health disorders (MHD) and substance use disorders (SUD). Various measures have been implemented to mitigate these adverse mental health consequences. Nevertheless, the significant rise in mental health challenges has underscored the role of psychotropic drug prescriptions as an important metric for assessing mental health trends in certain populations. During the pandemic, data from high-income countries, indicate an increase in the prescription of psychotropic drugs. This might reflect heightened mental health needs in the general population, but also may underscores the exacerbated vulnerability of individuals with pre-existing concurrent MHD/SUD. Prescribing psychotropic drugs can be complex due to the potential for misuse and the need for careful monitoring to balance therapeutic effects with the risks.

**Objectives:**

This study aimed to evaluate 1) the number of prescriptions of psychotropic drugs in persons with concurrent MHD and SUD in the years before and during the two COVID-19 pandemic years (2020 and 2021) and 2) to study the impact of the COVID-19 pandemic on the consumption of psychotropic drugs among this population.

**Methods:**

We conducted a retrospective cohort study based on a data file constructed by merging individual-level information from the Norwegian Patient Register and the Norwegian Prescribed Drug Registry, analysing data from 2019-2021. The International Classification of Diseases (ICD)-10 main diagnosis, chapter V have been used to identify persons with MHD/SUD. We recognised 35.000 individuals who received a diagnosis of co-occurring MHD/SUD between the years 2019-2021. A graphical approach and descriptives were applied to study the population. Interrupted time series analysis was used to compare changes in trends in the prescriptions dispensed by eight psychotropic drug classes, according to the consecutive COVID-19 waves during the first two years of the pandemic.

**Results:**

Preliminary analysis show, an increased prescription rate in all eight psychotropic drugs classes in persons with MHD/SUD during the first two years of the pandemic, compared to 2019. The largest increases were shown to appear parallel with the COVID-19 waves. Antipsychotics and anxiolytics were the most prevalent prescribed drugs, followed by opiates, hypnotics & sedatives and antidepressants. Being female and older age was associated with higher odds of receiving a prescription, regardless psychotropic drug class.

**Conclusions:**

The prescription of psychotropic drugs has gradually increased from 2019-2021. More research is needed to differentiate increases due to unmet needs versus overprescribing and further, to study the long-term effects of the pandemic regarding the utilization of psychotropic drugs in this vulnerable patient group.

**Disclosure of Interest:**

None Declared

